# Estimating the difference in prevalence of common mental disorder diagnoses for Aboriginal and Torres Strait Islander peoples compared to the general Australian population

**DOI:** 10.1017/S2045796022000233

**Published:** 2022-06-21

**Authors:** I. S. Page, A. J. Ferrari, T. Slade, M. Anderson, D. Santomauro, S. Diminic

**Affiliations:** 1 School of Public Health, University of Queensland, Brisbane, Australia; 2 Queensland Centre for Mental Health Research, Brisbane, Australia; 3 Institute for Health Metrics and Evaluation, University of Washington, Seattle, USA; 4 The Matilda Centre for Research in Mental Health and Substance Use, The University of Sydney, Sydney, Australia; 5 School of Psychology, University of Queensland, Brisbane, Australia

**Keywords:** Aboriginal and Torres Strait Islander, First Nations, mental disorder, social and emotional wellbeing

## Abstract

**Aims:**

There is currently little nationally representative diagnostic data available to quantify how many Aboriginal and Torres Strait Islander people may need a mental health service in any given year. Without such information, health service planners must rely on less direct indicators of need such as service utilisation. The aim of this paper is to provide a starting point by estimating the prevalence ratio of 12-month common mental disorders (i.e. mood and anxiety disorders) for Indigenous peoples compared to the general Australian population.

**Methods:**

Analysis of the four most recent Australian Indigenous and corresponding general population surveys was undertaken. Kessler-5 summary scores by 10-year age group were computed as weighted percentages with corresponding 95% confidence intervals. A series of meta-analyses were conducted to pool prevalence ratios of Indigenous to general population significant psychological distress by 10-year age groups. The proportion of respondents with self-reported clinician diagnoses of mental disorders was also extracted from the most recent survey iterations.

**Results:**

Indigenous Australians are estimated to have between 1.6 and 3.3 times the national prevalence of anxiety and mood disorders. Sensitivity analyses found that the prevalence ratios did not vary across age group or survey wave.

**Conclusions:**

To combat the current landscape of inequitable mental health in Australia, priority should be given to populations in need, such as Indigenous Australians. Having a clear idea of the current level of need for mental health services will allow planners to make informed decisions to ensure adequate services are available.

## Introduction

It is estimated that Aboriginal and Torres Strait Islander peoples (henceforth respectfully referred to as Indigenous) have inhabited Australia and its surrounds for over 65 000 years, making them one of the oldest cultures worldwide, with unrivalled history, resilience and strength (Dudgeon *et al*., 2014; Pascoe and Horton, 2018). Despite this, the consequences of European settlement and discriminatory policies can still be felt today, and the health of Indigenous Australians has been described by some as ‘Third World health in a First World nation’ (Carson, 2007). It has been estimated that Indigenous Australians, who now account for approximately 3.3% of the overall Australian population (Australian Institute of Health and Welfare, 2019), experience a burden of disease and injury 2.3 times that of non-Indigenous Australians (Australian Institute of Health and Welfare, 2021). Mental and substance use disorders account for the greatest proportion of this disease burden for Indigenous peoples (23%) (Australian Institute of Health and Welfare, 2021).

A study of Indigenous and non-Indigenous people in Victoria found that this difference is not inherent, but can be attributed to factors such socio-economic status, negative perceptions of the residential neighbourhood, lack of social support from family, social and civic distrust, age, sex, marital status, household composition and rurality (Markwick *et al*., 2015). When these factors were controlled for, the authors found that the health gap was rendered insignificant. Despite this, it is important to address the current landscape of inequitable mental health.

To reduce the burden of disease and close the health gap between non-Indigenous and Indigenous Australians, improved planning and service provision is needed. This should include a focus on Indigenous specific models of mental health care, such as those delivered through Aboriginal Community Controlled Health Organisations, which have shown to be effective at improving mental health outcomes (Panaretto *et al*., 2014; National Aboriginal Community Controlled Health Organisation, 2020). To plan and deliver these services effectively, it is helpful to have a clear understanding of the prevalence of mental disorders in Indigenous populations. Surprisingly, there is little nationally representative diagnostic data on mental disorders with which to estimate prevalence. In the absence of this, proxy measures can be used to estimate the prevalence of mental disorders in the population.

Limited availability of culturally appropriate diagnostic tools and insufficient resourcing to correctly sample Indigenous populations has contributed to the lack of robust, nationally representative prevalence data. There are mixed opinions as to the validity and utility of the main diagnostic classifications of mental disorders – the Diagnostic and Statistical Manual (DSM) (American Psychiatric Association, 2013) and International Classification of Diseases (ICD) (World Health Organization, 1992) – for Indigenous populations. As Indigenous cultures view mental health in a holistic way encompassing physical, social, emotional and spiritual wellbeing, these diagnostic measures are often viewed as being too limited in their ability to measure the full spectrum of social and emotional wellbeing (Littlefield and Dudgeon, 2010). Obtaining reliable prevalence estimates for small subpopulations also requires specific sampling methodology. For example, to achieve reliable estimates for Māori, Pacific and Asian ethnic groups, the New Zealand Health Survey used a dual-frame approach, combining overall area-based sampling with more targeted sampling of specific areas which have a higher concentration of Māori, Pacific and Asian residents (Ministry of Health, 2016). Unfortunately, this kind of sampling can be both time and resource intensive, and the Australian National Survey of Mental Health and Wellbeing (NSMHWB, last published in 2007) did not include sufficient Indigenous participants to ensure reliable estimates of mental disorder prevalence within Indigenous populations (Australian Bureau of Statistics, 2009).

There are some prevalence estimates for mood and anxiety disorders in Indigenous Australians available, however these are based on subsamples of the population and vary significantly. Previous work, including a systematic literature review, exploring the prevalence of mental disorders in Indigenous populations found the prevalence of mood and anxiety disorders to range between 7.7–43.1 and 17.2–58.6% respectively (Black *et al*., 2015; Nasir *et al*., 2018). The variability between studies was accounted for by sample type (e.g. community studies *v*. prison populations), diagnostic assessment (e.g. instrument used, diagnoses *v*. symptom scales) and timeframe (e.g. 12 month *v*. lifetime) (Black *et al*., 2015). Whilst these estimates provide some indications of the prevalence of common mental disorders within Indigenous Australian populations, none are nationally representative.

Although no national mental health surveys conducted to date have allowed for estimation of prevalence within Indigenous populations, there have been some mental health measures included in general health surveys. The National Aboriginal and Torres Strait Islander Health Survey (NATSIHS) and National Aboriginal and Torres Strait Islander Social Survey (NATSISS) are regularly conducted to assess the health of Indigenous Australians. Although diagnostic tools are not included, these nationally representative population surveys provide estimates of the prevalence of self-reported clinical diagnoses of mental disorders (e.g. answering yes to questions like ‘Have you ever been diagnosed with depression by a health professional?’) and significant psychological distress (as measured by the Kessler Psychological Distress Scale; K-5), both of which can be used as proxy measures for common mental disorders. In an Australian general population sample the K-10 has been found to be correlated with structured diagnostic interview diagnoses of mood and anxiety disorders (Andrews and Slade, 2001*a*). The K-5 measure used in the NATSIHS and NATSISS is an adapted version of the K-10, specifically for use in Indigenous Australian populations. One study assessed the agreement of K-10 and K-5 distress scores for Indigenous Australians aged over 45 years and found the K-5 yielded similar distress scores to the K-10 (McNamara *et al*., 2014).

As neither self-reported clinical diagnosis nor psychological distress are gold standards for quantifying the prevalence of mental disorders, one available method is to consider the magnitude of differences in the overall prevalence of these measures between Indigenous peoples and the general population. The aim of this study was to estimate the prevalence ratio of common mental disorders between Indigenous peoples and the general population, with the ultimate purpose of better understanding the mental health service needs of Indigenous populations. Prevalence ratios (prevalence in an Indigenous sample divided by prevalence in a general population sample) using available indicators of mental ill health such as self-reported clinical diagnosis and psychological distress can then be applied to known national prevalence estimates of common mental disorders to approximate the estimated national prevalence of common mental disorders for Indigenous Australians. These prevalence estimates can be used to better understand mental health service needs for this population.

## Methods

### Survey and sample

The prevalence of self-reported clinical diagnosis and psychological distress for Indigenous populations was drawn from multiple iterations of the NATSIHS and NATSISS, and for the general population from multiple iterations of the National Health Survey (NHS). The NATSIHS and NATSISS are nationally representative cross-sectional household surveys of Indigenous peoples' health which are alternately completed approximately every 4 years. To gain a representative sample, a multi-stage sampling process is used, consisting of a community sample (made up of discrete Indigenous communities) and a non-community sample (made up of private dwellings outside Indigenous communities) (Australian Bureau of Statistics, 2019*b*). Total sample sizes for the past four iterations have ranged from approximately 9000 (2012/13 NATSIHS) to over 13 000 people (2008 NATSISS) and overall response rates have ranged from 73% (2018/19 NATSIHS) to 83% (2008 NATSIHS).

The NHSs are nationally representative cross-sectional household surveys of general population health completed approximately every 3 years. Dwellings are selected at random using a multistage area sample of private dwellings and one adult and one child from each dwelling is randomly selected for inclusion in the survey (Australian Bureau of Statistics, 2018). Whilst Indigenous peoples are not excluded from this survey, people residing in Indigenous communities and very remote areas of Australia were not part of the sampling frame. Similar to the NSMHWB, the NHSs did not include sufficient Indigenous respondents to generate reliable estimates specific to Indigenous populations.

The NATSIHS, NATSISS and NHS are all conducted by the Australian Bureau of Statistics (ABS), and interviews are conducted by trained ABS interviewers. The ABS provides person weights reflecting the age and sex distribution of the Australian population alongside the data files for analysis. The survey methodologies for the NATSIHS, NATSISS and NHS are explained in detail elsewhere (Australian Bureau of Statistics, 2010, 2018, 2019*b*). See Table 1 for details of the surveys included in this study.

**Table 1.** Nationally representative household surveys included in this study

Survey	Response rate	Sample size included in this study (persons)		Corresponding general population survey
		K-5^a^	Self-reported clinical diagnosis	
Aboriginal and Torres Strait Islander surveys				
NATSISS 2008^b^	78–83%^c^	7053	–	NHS 2007/08
NATSIHS 2012/13^d^	74–77%^c^	5419	–	NHS 2011/12
NATSISS 2014/15^e^	77–78%^c^	6545	–	NHS 2014/15
NATSIHS 2018/19^f^	73%	6209	10 579	NHS 2017/18
General population surveys				
NHS 2007/08^g^	91%	15 770	–	–
NHS 2011/12^h^	85%	15 387	–	–
NHS 2014/15^i^	82%	14 477	–	–
NHS 2017/18^j^	76%	15 861	21 315	–

a Respondents aged over 18 years were asked the K-5 questions; those who had more than one K-5 item missing were excluded from analysis (0.1–3.3% excluded).

b National Aboriginal and Torres Strait Islander Social Survey 2008 (Australian Bureau of Statistics, 2008)

c A range is provided because there were differing response rates for different modules of the survey.

d National Aboriginal and Torres Strait Islander Health Survey 2012/13 (Australian Bureau of Statistics, 2013)

e National Aboriginal and Torres Strait Islander Social Survey 2014/15 (Australian Bureau of Statistics, 2015a)

f National Aboriginal and Torres Strait Islander Health Survey 2018/19 (Australian Bureau of Statistics, 2020)

g National Health Survey 2007/08 (Australian Bureau of Statistics, 2008)

h National Health Survey 2011/12 (Australian Bureau of Statistics, 2012b)

i National Health Survey 2014/15 (Australian Bureau of Statistics, 2015b)

j National Health Survey 2017/18 (Australian Bureau of Statistics, 2018a)

### Measures

#### Self-reported clinical diagnoses of mental disorder

The self-reported diagnostic module included in the 2018/19 NATSIHS and 2017/18 NHS contained a set of questions on mental and behavioural conditions asked for all respondents aged 2 years and over (proxy interviews with a parent or guardian were conducted for children aged less than 15 years) (Australian Bureau of Statistics, 2019*a*). Using comparable items from both surveys, respondents were classified as having a self-reported clinical diagnosis of mental disorder if they reported being diagnosed with a mood or anxiety disorder by a health professional, and it was current in the past 12 months. The disorders included from each survey were: depression, manic episode, bipolar affective disorder, other mood (affective) disorders and anxiety disorders (including generalised anxiety disorder, panic disorder, panic attacks, phobic anxiety disorders, obsessive-compulsive disorder, post-traumatic stress disorder and other anxiety-related disorders). As the diagnostic module included in the surveys has changed over each survey wave, only the most recent NATSIHS and NHS iterations were analysed.

#### Significant psychological distress (Kessler scales)

The K-10 was developed in 1992 by Kessler *et al*. (2002) for use in the United States National Health Interview Survey as a brief measure of non-specific psychological distress. The K-10 comprises ten questions (see Table 2) and measures the frequency of each in the past 30 days. The responses to the K-10 are provided on a five-item scale for each question: all of the time (5), most of the time (4), some of the time (3), a little of the time (2) and none of the time (1). The scores are then summed to create an overall score ranging from 10 to 50 (Andrews and Slade, 2001*b*). Levels of psychological distress can be categorised into the following groups: low (10–15), moderate (16–21), high (22–29) and very high (30–50). A six-item version of the K-10 has also been widely used and is referred to as the Kessler High Distress Measure (K-6). When the first NATSIHS (2004/05) questionnaire was being developed, stakeholders modified this measure to ensure appropriateness for Indigenous Australians. Specifically, one item was removed, and some slight wording changes were made to two items to enhance understanding in an Indigenous context (Australian Institute of Health and Welfare, 2009*b*). K-5 scoring is the same as for K-10 – scores are summed to create an overall score ranging from 5 to 25. Levels of psychological distress can be categorised into the following groups: low (5–7), moderate (8–11), high (12–14) and very high (15+). All surveys included in this study used the K-5 or K-10. Where K-10 was collected (in the NHS), for comparability purposes, the subset of questions equivalent to the K-5 measure was extracted for this study.

**Table 2.** Comparison of K-5 and K-10 questionnaires, differences in wording of questions underlined (based on ABS Table C: K-5 comparison with relevant K-10 questions (Australian Bureau of Statistics, 2012a))

Item	K-5^1^	Item	K-10^2^
	In the last 4 weeks, about how often did you feel…		In the past 4 weeks, about how often did you feel…
		1	tired out for no good reason?
1	nervous?	2	**nervous?**
		3	so nervous that nothing could calm you down?^c^
2	without hope?	4	**hopeless?**
3	restless or jumpy?	5	**restless or fidgety?**
		6	so restless that you could not sit still?^c^
		7	depressed?
4	everything was an effort?	8	**that everything was an effort?**
5	so sad that nothing could cheer you up?	9	**so sad that nothing could cheer you up?**
		10	**worthless?**

K-6 items are in bold.

a Version used in 2018/19 NATSIHS survey (Australian Bureau of Statistics, 2019)

b Version used in 2017/18 NHS survey (Australian Bureau of Statistics, 2018b)

c These questions are not asked if the response to the preceding question was ‘none of the time’ (Andrews and Slade, 2001)

### Data analysis

Each included survey (see Table 1) was analysed individually in an online data analysis facility that allows for statistical analysis of highly confidential data using Stata (Stata-MP version 16.0), taking into account the complex survey design and weighting procedures. The jackknife method was employed to compute standard errors (Bell, 2000). The K5 summary scores (in four groups – low, medium, high, very high; and in two groups – low/medium; high/very high) were computed using weighted percentages and 95% confidence intervals. If a respondent had one item missing, then the missing item was imputed using an average of the four other items (<1% for all surveys). If the respondent had more than one item missing, they were excluded from analysis. The weighted prevalence (with 95% confidence intervals) of respondents with self-reported clinician diagnoses of mental disorders was also computed. Prevalence was estimated by 10-year age groups (18–24, 25–34, 35–44, 45–54, 55–64, 65+) to control for age variation between the two population groups of interest. Results were then combined into a master dataset and further analysed using R (R version 3.5.1). Random-effects meta-analysis was used to pool prevalence ratios of Indigenous to general population K-5 scores using two different cut-off points (‘high/very high’ and ‘very high’) by 10-year age groups. A sensitivity analysis was conducted to determine whether the K-5 prevalence ratios varied significantly across different age groups, or surveys. This was achieved via meta-regression with mid-age (midpoint between minimum and maximum age of the estimates) and mid-year (midpoint between earliest and latest year of the survey wave) as covariates.

## Results

**Fig. 1.**
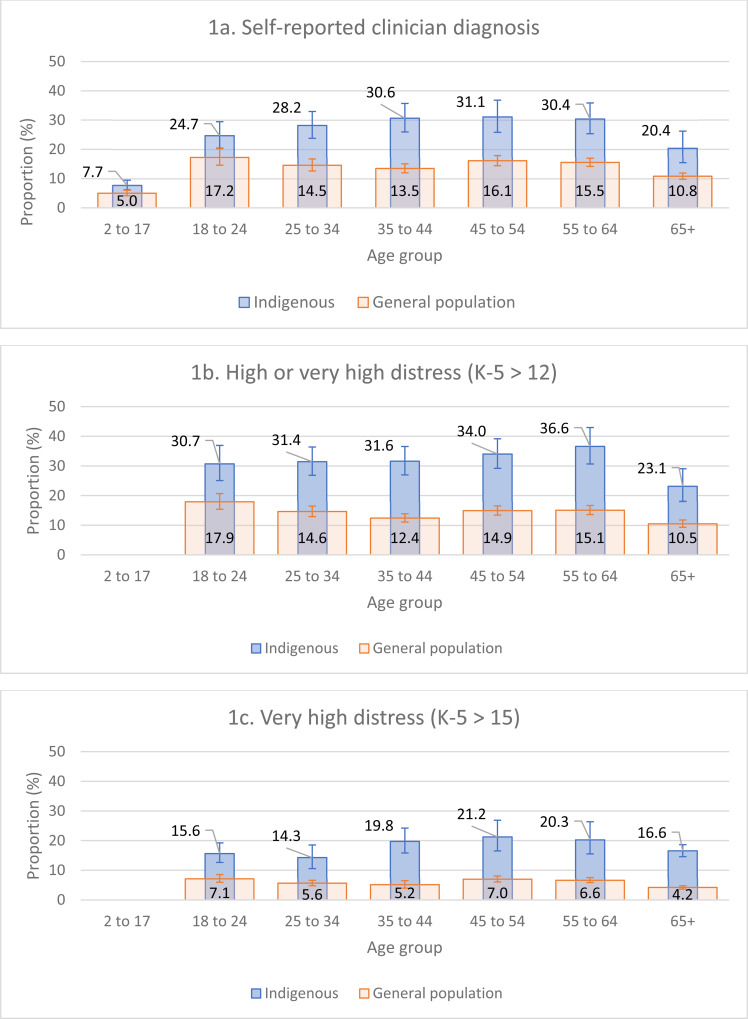
Proportion of Indigenous (2018/19 NATSIHS) and general population (2017/18 NHS) with (a) current self-reported clinician diagnosis; (b) high or very high psychological distress (K-5 score >12); and (c) very high psychological distress (K-5 score >15), by 10-year age groups.

Figure 1 shows the proportion of Indigenous and general population respondents from the most recent survey waves (2018/19 NATSIHS and 2017/18 NHS) who reported (a) having a current self-reported clinical diagnosis of a mood or anxiety disorder; (b) having high or very high psychological distress and (c) having very high psychological distress. The proportion of respondents with current diagnoses varied across age group and sample. For the Indigenous sample, the proportion of respondents with a self-reported diagnosis ranged from 7.7% (2–17 years) to 31.1% (45–54 years) (Fig. 1). In comparison, for the general population sample, this was 5.0% (2–17 years) to 17.2% (18–24 years). The proportion of respondents with high or very high psychological distress ranged from 23.1% (65+ years) to 36.6% (55–64 years) for the Indigenous sample and from 10.5% (65+ years) to 17.9% (18–24 years) in the general population. The proportion of respondents with very high psychological distress ranged from 14.3% (25–34 years) to 21.2% (45–54 years) for the Indigenous sample and from 4.2% (65+ years) to 7.1% (18–24 years) in the general population.

Across all age groups and all indicators there was a significant difference between Indigenous and general population respondents' scores, as judged by non-overlapping confidence intervals. Similar proportions were found across all survey waves; see Online Resource 1 for all psychological distress scores by survey wave and Indigenous status.

Rates of self-reported mood or anxiety disorder diagnosis in Indigenous peoples were 1.6 times that for the general population (Table 3). Psychological distress was 2.5 (high/very high) to 3.3 times (very high) higher for Indigenous people. Whilst the prevalence ratios differed slightly across different 10-year age groups, overlapping confidence intervals indicate that this variation was not statistically significant. Sensitivity analyses found neither age group (*β* ≤ 0.001; *p* = 0.746) nor survey wave (*β* = 0.012; *p* = 0.081) had a statistically significant impact on the prevalence ratios for psychological distress. See Online Resource 2 for forest plots of the three meta-analyses.

**Table 3.** Meta-analysis prevalence ratios (95% confidence intervals) for Indigenous Australians compared to the general Australian population by 10-year age groups

	Self-reported clinical diagnosis^a^	High/very high K-5	Very high K-5
Under 18	1.5 (1.2–1.8)	–	–
18–24	1.3 (1.0–1.6)	2.1 (1.8–2.5)	2.7 (2.2–3.2)
25–34	1.7 (1.4–2.1)	2.4 (2.2–2.6)	3.2 (2.7–3.8)
35–44	1.9 (1.6–2.2)	2.8 (2.6–3.0)	4.0 (3.5–4.5)
45–54	1.6 (1.3–1.9)	2.6 (2.3–2.9)	3.3 (2.9–3.7)
55–64	1.7 (1.4–2.0)	2.6 (2.4–2.9)	3.4 (2.9–3.9)
65+	1.4 (1.1–1.8)	2.4 (2.1–2.7)	3.1 (2.5–3.8)
All ages (2+ years)	1.6 (1.5–1.7)	–	–
All ages (18+ years)	1.6 (1.4–1.8)	2.5 (2.4–2.6)	3.3 (3.1–3.5)

a 2018/19 NATSIHS and 2017/18 NHS data only.

## Discussion

This study estimates that Indigenous peoples in Australia have 1.6–3.3 times greater prevalence of common mental disorder diagnoses than the general Australian population. This large range is due to differences in the indicators used to estimate prevalence (in the absence of representative survey data on the prevalence of diagnosable mental disorders). Using self-reported diagnosis results in a lower prevalence ratio, whereas K-5 scores lead to a higher ratio (indicating a greater difference in estimated prevalence). None of these prevalence ratios should be used without consideration of the pros and cons of each approach.

Self-reported diagnostic prevalence estimates require the participants to (1) have consulted a health practitioner, (2) be diagnosed with a mental disorder and (3) for that diagnosis to be formally recorded, communicated to them and clearly understood and recalled by them. The 2007 NSMHWB found that approximately one-third (34.9%) of people with a mental disorder used services for mental health problems in the past 12 months (Burgess *et al*., 2009). This indicates that a large proportion of people with a mental disorder may not be accessing services; however, service availability and access has been increasing over time (Jorm *et al*., 2017). Service access also varies by Indigenous status, and current levels of care are seen by many as inadequate and inequitable (Dudgeon *et al*., 2020). This has been attributed to a range of barriers including costs, experiences of racism and poor communication with healthcare workers (Aspin *et al*., 2012). Because of this, the likelihood for an Indigenous person to see a professional may be lower.

Additionally, the likelihood of a clinician making a diagnosis cannot be assumed to be consistent across Indigenous and non-Indigenous populations (Adams *et al*., 2014). Few studies have directly compared self-reported prevalence with clinician diagnosis. However, a Spanish study conducted by Sanchez-Villegas *et al*. (2008) found that out of 62 participants who had self-reported diagnoses of depression, 42 (74.2%) of those were confirmed by a clinician's official diagnosis (true positive). On the other hand, out of 42 participants who did not self-report a depression diagnosis, 34 (81.0%) were confirmed to be depression-free (true negative), indicating a higher proportion of true negatives than true positives. It is unclear whether similar results would be found in an Indigenous sample.

The accuracy of self-reported diagnostic prevalence estimates can be further explored by comparing diagnostic and self-reported data from different samples. Nationally representative diagnostic data for Indigenous Australians is unavailable; however, the 2007 NSMHWB found diagnostic prevalence of common mental disorders for the general Australian population to be 20.0% for 12-month conditions, and 45.5% for lifetime diagnoses (Slade *et al*., 2009). In the same year, the 2007/08 NHS found only 11.2% of respondents reported having a long-term mental or behavioural condition that was identified by a mental health professional (Australian Institute of Health and Welfare, 2009*a*). Interestingly, the rate of self-reported mental health conditions was significantly higher in the most recent NHS survey, which recorded that one in five (20.1%) Australians had a mental or behavioural condition (Australian Bureau of Statistics, 2018). This may reflect changes in both mental health literacy and service access over time; a South Australian study found that there was a significant increase in mental health literacy related to depression between 1998 and 2004 (Goldney *et al*., 2005). It may also reflect significant increases in treatment rates of mental disorders since 2007, largely attributed to the introduction of subsidised mental health service initiatives by the Australian government (Jorm *et al*., 2017). Although the more recent self-reported prevalence estimates of mental disorders (from the 2018/19 NHS) are more aligned with the estimates from the diagnostic health survey in 2007, it is important to note they are not directly comparable due to differing survey methodologies including the disorders included, and year of data collection.

Significant psychological distress, as measured using the K-10, is correlated with diagnoses of mood and anxiety disorders in the Australian general population (Andrews and Slade, 2001*a*). Although no similar analysis has been done to assess K-5 for Indigenous peoples, high or very high levels of psychological distress have been used as a proxy measure for common mental disorders in this study. The prevalence ratio for respondents with high/very high psychological distress was lower (2.4) than that for the higher threshold for respondents with very high psychological distress only (3.1). This may be a reflection of the severity distribution of mental health problems among Indigenous populations – i.e. a greater number of Indigenous Australians have higher levels of distress than the general population. This is shown by the ratio getting larger as Indigenous peoples are more distressed relative to the general population.

### Strengths and limitations

This study used robust, nationally representative survey data (compared to previous prevalence studies, for example) to estimate the prevalence ratio of common mental disorders between Indigenous peoples and the general population. This was done using multiple indicators, all of which showed a substantially higher rate of mental ill-health in Indigenous peoples. The main limitation to this study is the use of proxy measures (rather than diagnostic tools) to determine prevalence ratios. In the absence of other data however, these are the best measures available, and previous studies have found these measures to be correlated with common mental disorders (Andrews and Slade, 2001*a*).

There have been five NATSIHS/NATSISS iterations completed to date, and it was expected that all of these would be included in this analysis. However, data from the first NATSIHS (2004/05) were unable to be included, as the dataset available was structured differently to the more recent iterations, meaning it could not be analysed in a consistent way. Despite this, 11 years of data were included, and it was found that across those four iterations survey wave did not have a significant impact on prevalence ratios.

The response rates for included surveys ranged from 73 to 83%; this is a significant strength, considering that many population health surveys have much lower response rates (e.g. the 2007 NSMHWB response rate was 60% (Slade *et al*., 2009)). Despite this, the NHSs consistently had higher response rates than the corresponding NATSIHSs or NATSISSs. It has previously been found that survey non-responders have poorer mental health than responders (de Graaf *et al*., 2010; Torvik *et al*., 2011). If this is true for the non-responders across the included surveys, the prevalence estimates may be more of an underestimate for the Indigenous surveys than the general population surveys (because of the higher response rates for the general population surveys). It is unclear whether this possible bias has affected prevalence ratios. For the significant psychological distress measure (K-5), any respondent who had more than one item score missing was excluded from analysis. Whilst this may have resulted in bias, in practice only a small proportion of each survey sample were excluded (ranging from 0.1 to 3.3%).

It is important to note that Indigenous peoples are very diverse (Ogilvie *et al*., 2021), and true prevalence of common mental disorders within different communities may vary. This study provides the template from which future analysis on subnational data may be undertaken, which would be appropriate if planning for smaller populations. Furthermore, the findings from this study can be validated using more robust measures once data from the upcoming national Indigenous mental health survey (Australian Government Department of Health, 2020) become available. Finally, as these data range from 2007 to 2019, it also provides a baseline from which future data capturing prevalence ratios or distress levels related to the Covid-19 pandemic could be measured. Continuous monitoring in this way is important to evaluate any change to this disparity.

## Conclusions

To combat the current landscape of inequitable mental health and mental health service access in Australia, priority should be given to populations in need. Having a better idea of the prevalence of common mental disorders within Indigenous populations will allow planners to make informed decisions to ensure adequate services are available. The prevalence ratios from this study can be applied to national prevalence data to assist with this. For example, the most recent national survey of mental health and wellbeing (2007 NSMHWB) found that the prevalence of 12-month mood, anxiety and total common mental disorders to be 6.2, 14.4 and 20%, respectively (Slade *et al*., 2009). Based on the prevalence ratio of 1.6–3.3, it could then be estimated that, in 2007, the prevalence of mood, anxiety and total common mental disorders for Indigenous populations nationally might be 9.9–20.5, 23.0–47.5 and 33.0–68.0%, respectively. These estimates are within the range of estimates from previous studies that were based on subsamples of the population using varying sample types, assessments and timeframes (Black *et al*., 2015; Nasir *et al*., 2018).

The study highlights enduring higher levels of mental ill-health for Indigenous peoples across all age groups over 11 years. It is important to reiterate the determinants which contribute to observable deficits in mental health outcomes for Indigenous peoples are *not* inherent but are due to historical factors leading to the current landscape in Australia (Markwick *et al*., 2015). This study did not seek to control for these factors, as doing so would not give an accurate indication of the current levels of mental health service needs.

Closing these gaps requires a holistic approach to care, attention to primary prevention and improved access to effective and culturally appropriate social and emotional wellbeing services (Page *et al*., 2022). In addition to ensuring adequate and appropriate services are available, it is important to improve service utilisation. Identified keys to doing this include viewing mental health holistically as social and emotional wellbeing, having Indigenous identified staff and incorporating relationships with land and family into care (Dingwall and Cairney, 2010; Berry and Crowe, 2014).

## Data Availability

The datasets analysed during the current study are available in the ABS DataLab secure environment. To access these data, you need to be an approved researcher with a login who has undertaken ABS DataLab training. More information is available here: https://www.abs.gov.au/ausstats/abs@.nsf/mf/1406.0.55.007.

## Supplementary material

For supplementary material accompanying this paper visit http://doi.org/10.1017/S2045796022000233. click here to view supplementary material
