# Supplementary material for: Estimating the difference in prevalence of common mental disorder diagnoses for Aboriginal and Torres Strait Islander peoples compared to the general Australian population
Source: Epidemiol Psychiatr Sci. 2022 Jun 21;31:e44. doi: 10.1017/S2045796022000233 (PMC9228582; doi:10.1017/S2045796022000233)
Supplement: Supplementary file 1 [file epssup.zip › S2045796022000233sup001.docx]

# Online resource 1: High/very high and very high K-5 scores by survey year and age group
