# Supplementary material for: Estimating the difference in prevalence of common mental disorder diagnoses for Aboriginal and Torres Strait Islander peoples compared to the general Australian population
Source: Epidemiol Psychiatr Sci. 2022 Jun 21;31:e44. doi: 10.1017/S2045796022000233 (PMC9228582; doi:10.1017/S2045796022000233)
Supplement: Supplementary file 1 [file epssup.zip › S2045796022000233sup002.docx]

# Online resource 2: Forest plots from meta-analyses


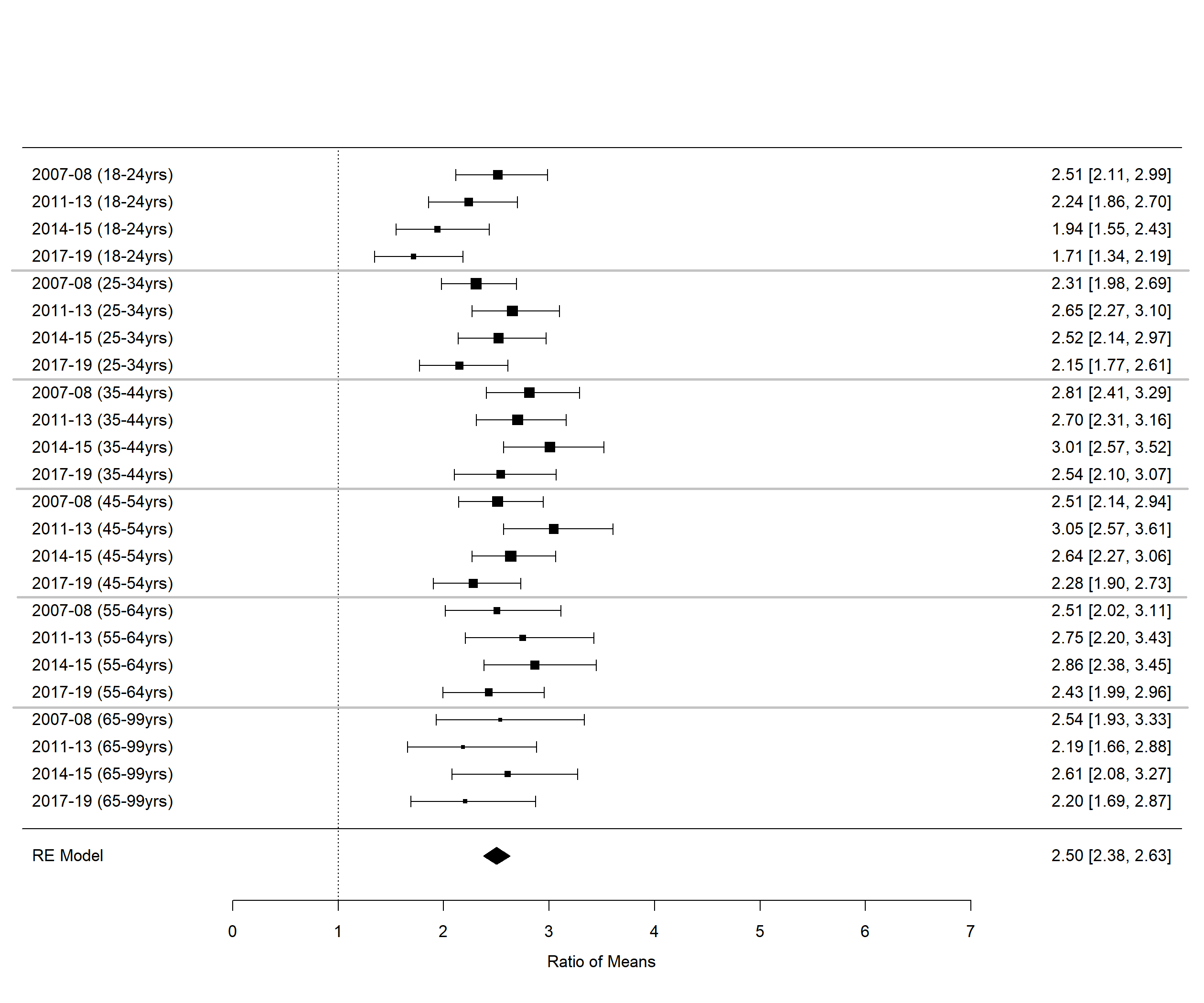


Figure 1 Forest plot of all ages meta-analysis of high/very high K5 scores combining all four survey pairs


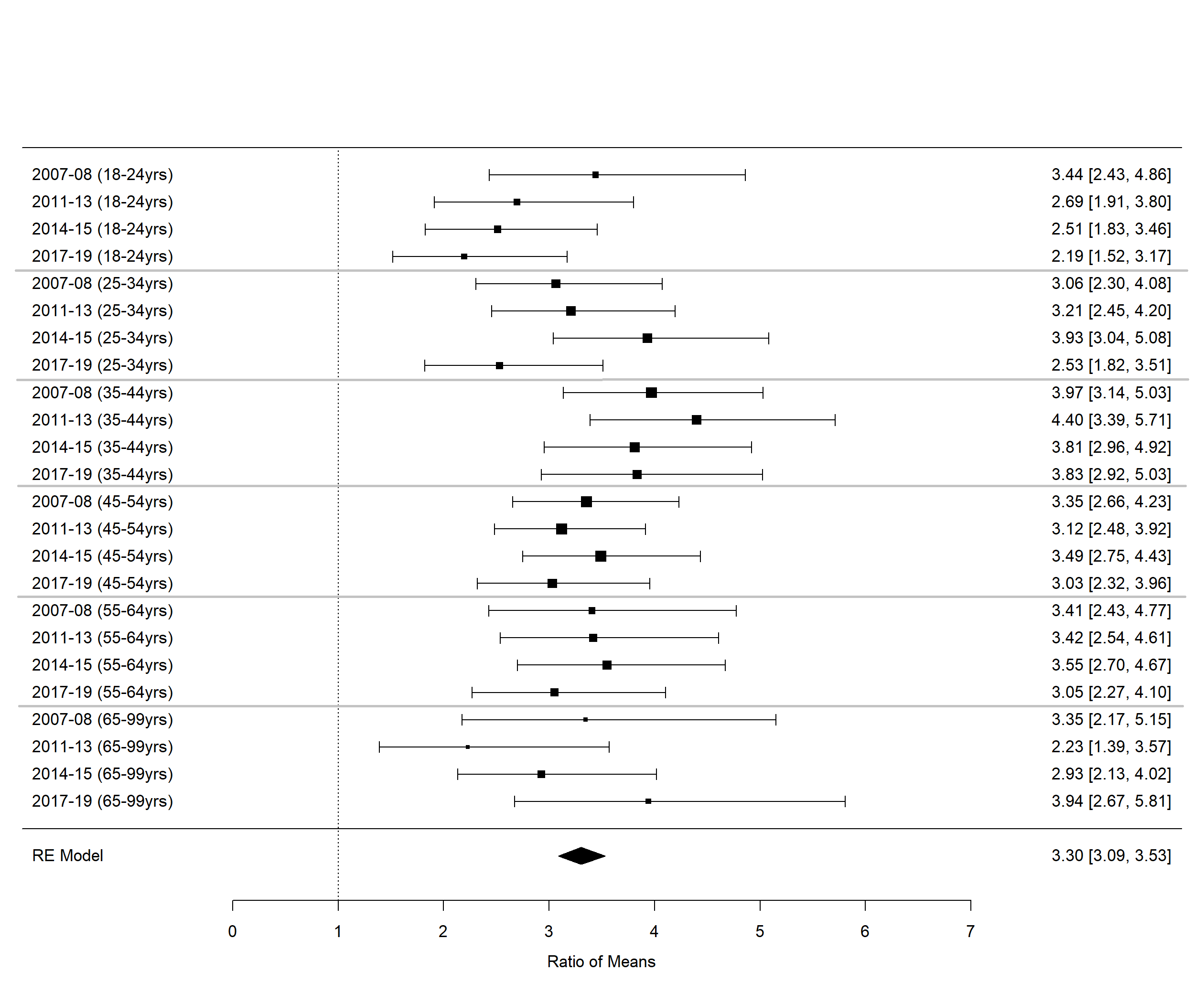


Figure 2 Forest plot of all ages meta-analysis of very high K5 scores combining all five survey pairs


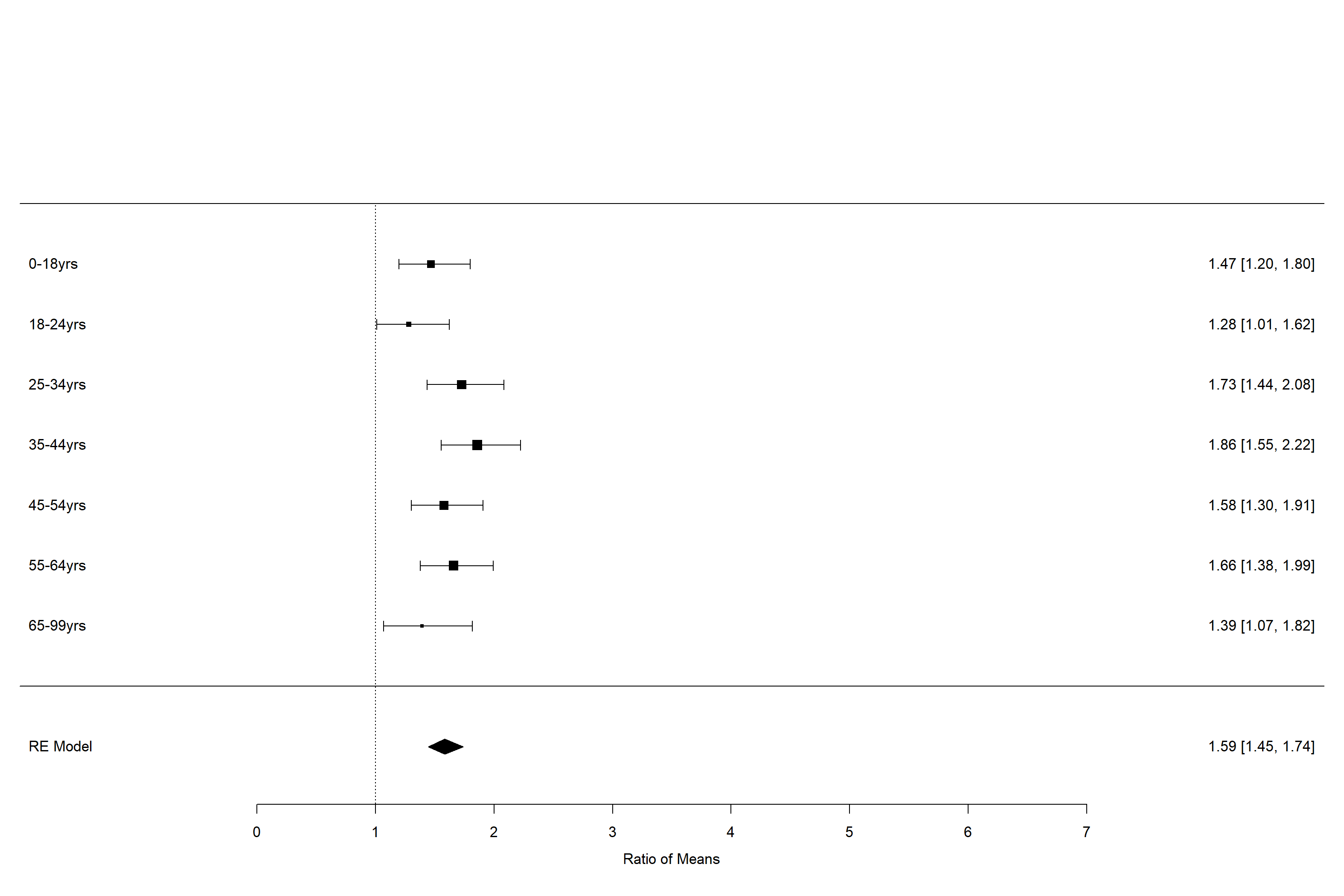


Figure 3 Forest plot of all ages meta-analysis of self-reported diagnosis in NATSIHS 2018/19 and NHS 2017/18 surveys
